# Understory Bird Communities in Amazonian Rainforest Fragments: Species Turnover through 25 Years Post-Isolation in Recovering Landscapes

**DOI:** 10.1371/journal.pone.0020543

**Published:** 2011-06-22

**Authors:** Philip C. Stouffer, Erik I. Johnson, Richard O. Bierregaard, Thomas E. Lovejoy

**Affiliations:** 1 School of Renewable Natural Resources, Louisiana State University Agricultural Center and Louisiana State University, Baton Rouge, Louisiana, United States of America; 2 Biology Department, University of North Carolina, Charlotte, North Carolina, United States of America; 3 The Heinz Center, Washington, District of Columbia, United States of America; 4 Projeto Dinâmica de Fragmentos Florestais, Instituto Nacional de Pesquisas da Amazônia and Smithsonian Tropical Research Institute, Manaus, Brazil; University of Hull, United Kingdom

## Abstract

Inferences about species loss following habitat conversion are typically drawn from short-term surveys, which cannot reconstruct long-term temporal dynamics of extinction and colonization. A long-term view can be critical, however, to determine the stability of communities within fragments. Likewise, landscape dynamics must be considered, as second growth structure and overall forest cover contribute to processes in fragments. Here we examine bird communities in 11 Amazonian rainforest fragments of 1–100 ha, beginning before the fragments were isolated in the 1980s, and continuing through 2007. Using a method that accounts for imperfect detection, we estimated extinction and colonization based on standardized mist-net surveys within discreet time intervals (1–2 preisolation samples and 4–5 post-isolation samples). Between preisolation and 2007, all fragments lost species in an area-dependent fashion, with loss of as few as <10% of preisolation species from 100-ha fragments, but up to 70% in 1-ha fragments. Analysis of individual time intervals revealed that the 2007 result was not due to gradual species loss beginning at isolation; both extinction and colonization occurred in every time interval. In the last two samples, 2000 and 2007, extinction and colonization were approximately balanced. Further, 97 of 101 species netted before isolation were detected in at least one fragment in 2007. Although a small subset of species is extremely vulnerable to fragmentation, and predictably goes extinct in fragments, developing second growth in the matrix around fragments encourages recolonization in our landscapes. Species richness in these fragments now reflects local turnover, not long-term attrition of species. We expect that similar processes could be operating in other fragmented systems that show unexpectedly low extinction.

## Introduction

Area effects on species richness in habitat fragments have been well studied, even if results have not been consistent [1], [2]. In recent years, emphasis has moved away from a viewing habitat fragments in a classic island biogeography context toward explicitly considering matrix effects and landscape-level habitat loss [3], [4], [5], [6]. Despite this recognition, however, most conclusions about patterns of species richness in fragments continue to be based upon short-term, ‘snapshot,’ samples that can’t recreate the dynamics of the system following isolation. We know little about the pace of extinctions following isolation, about the role of recolonization in maintaining or adding species, or about the stability of reduced communities in fragments. These are important questions, especially for evaluating the extinction debt, or difference between current and ultimate species richness, in habitat fragments [7], [8], [9]. For example, we need to know if documented species loss in fragments represents the start of a trajectory of extinctions or just the loss of a distinct subset of highly vulnerable species [1].

Insights into temporal processes for birds in fragments come from several studies, although lack of simultaneous temporal and spatial replication in most studies makes dissection of extinction/colonization dynamics difficult. An increasing number of long-term studies have shown loss of forest species from forest fragments, deforested landscapes, or semi-isolates, usually by comparing historical records such as checklists with results of contemporary surveys [10], [11], [12], [13], [14], [15]. Extinctions in these systems can be staggering, such as 60% of resident forest species lost from Bogor Botanical Gardens (BBG), an 86 ha urban woodlot on Java with extensive management and human use [16]. Robinson [13] found continued extinctions through 85 years following isolation on Barro Colorado Island (BCI), a 1600 ha hilltop isolated by creation of the Panama Canal, with about 20% of forest species now extinct. Robinson predicted the eventual loss of another subset of species represented at the time of his study by few individuals, but also noted the recolonization of about 5% of species previously thought to be extinct.

Work on shorter timescales, but involving repeated sampling with consistent methods, has revealed some temporal processes in more detail. Research with birds identified the crowding effect, a temporary increase in abundance or species richness in newly-isolated fragments due to displaced animals seeking refuge, which has also been observed in other taxa [17], [18], [19]. Borgella and Gavin [20] estimated extinction and colonization in Costa Rican forest fragments of 0.5–20 ha over a five year interval. They found both extinction and colonization in even the smallest fragments in their mixed agricultural landscape, some 40–50 years following fragmentation. Their study included an analytical improvement over the checklist/resurvey studies- they explicitly considered species present but not detected. This allows inferences from standardized sampling that is assumed to be incomplete [21], [22]. Certainly such an approach could be important in evaluating extinction/colonization dynamics over longer time scales, as it allows parameter estimation without perfect detection. Even more importantly, this technique can be used to examine species loss from formerly continuous forest following isolation, allowing an empirical estimate of extinction for a given site. Occupancy modeling approaches have recently expanded to multiple species [23], [24], and may eventually allow examination of temporal trends in diverse communities in which many species are rarely detected.

Island studies, including those from real islands, like BCI, and those from habitat islands isolated by long distances and permanently inhospitable matrix, like BBG, have been vital to developing a framework for studying habitat fragments. At the same time, most fragments exist in a dynamic landscape setting that profoundly affects processes, including extinction and colonization, within fragments [1], [6]. Examining long-term patterns of extinctions and colonizations of fragments in characteristic anthropogenic landscapes will help us to predict the long-term prospects for biodiversity in fragments, and will be a step toward a more holistic understanding of how biodiversity might be maintained where second growth and fragments replace unbroken forest [25], [26], [27], [28].

### The Biological Dynamics of Forest Fragments Project

The Biological Dynamics of Forest Fragments Project (BDFFP), a *terra firme* forest site near Manaus, Brazil (2° 30′S, 60°W), provides long-term history of species present in forest fragments. Eleven continuous forest plots were sampled beginning in 1979 and then isolated as 1-ha (*n* = 5), 10-ha (*n* = 4), and 100-ha (*n* = 2) fragments beginning in 1980 (see [29], [30] for details of the site selection and isolation processes). The fragments are spread over three large farms, all of which include primary forest connected beyond the farms' borders to vast areas of almost unbroken forest, particularly to the north. The fragments are 70–800 m from continuous forest. In the first years after isolation, the matrix included cattle pasture and areas abandoned after cutting. Over time, the amount of active pasture has decreased, and more and more area has been abandoned to second growth. As of 2007, all fragments were connected to primary forest by second growth at least six years old.

Birds at the BDFFP have been sampled systematically with mist nets since 1979. In summary, understory bird diversity and abundance declined dramatically after isolation in 1- and 10-ha fragments, although the decline generally followed a brief influx of individuals presumably responding to local forest clearing [17]. Following isolation, recovery in bird abundance largely tracked second growth dynamics, with increasing bird abundance during periods of uninterrupted matrix growth, but reduced abundance following second growth cutting that resulted in reisolation of the fragments [31], [32]. Patterns in 100-ha fragments have been more difficult to generalize because only one 100-ha fragment (fragment 3304) was isolated with the other 1- and 10-ha fragments; a second 100-ha fragment (2303) was isolated in 1990.

Extinction dynamics of birds over several time intervals have been examined in previous publications. Ferraz et al. [33] modelled extinctions from preisolation through 1993 with four approaches, three of which could be extended over time. Stratford and Stouffer [34] used field sampling to demonstrate area and isolation effects on terrestrial insectivores through 1995. Data from the next round of mist net sampling, in 2000–2001, were augmented with extensive field surveys for species that had been present in the 1993 sample [35]. Extinctions occurred in all fragments between 1992 and 2000, but most fragments showed a net gain in species, thus supporting only the model from Ferraz et al. [33] that included recolonization.

### Objectives

We now have another complete mist-net sample, from 2007, that has not been analyzed. Here we consider mist net samples from 1979–2007 to evaluate the long- term pattern of extinction and colonization in the fragments. We use a jackknife estimator that accounts for species present but not captured [21], [22], [36]. This method produces estimates of the proportion of species that go extinct or colonize between sets of samples, but provides no information on individual species. To examine the fate of individual species, we compare the preisolation net sample with extensive surveys from 2000 and 2007 that included standardized net samples plus additional field techniques to maximize the detection of species that were present. Based on these data, we asked the following: At ∼25 years after isolation, how many species are locally extinct in fragments, and how does extinction vary with fragment size? Which species are most likely to go extinct? Are the current communities in the fragments a result of long-term decay in species richness or a balance of extinction and colonization? How have extinction and colonization rates changed over time? Does reisolation of fragments lead to an increase in extinction and a decrease in colonization?

## Methods

### Ethics statement

Animal care protocols were approved by CEMAVE and IBAMA in Brazil (CNPq Processo EXC 021/06-C) and Louisiana State University Agriculture Center (IACUC A2006-02).

### Mist net sampling

Mist net samples come from lines of 8 or 16 nets (NEBBA type ATX, 36-mm mesh, 12×2 m) set up in continuous lines along established trails through the interior of delineated reserves that were then isolated (see [32] for more details of the sampling protocol). We used a single line of 8 nets in 1-ha fragments, a single line of 16 nets in 10-ha fragments, and 3 lines of 16 nets in 100-ha fragments and continuous forest. Each line was netted for one day at a time from 0600-1400. Fragments were generally sampled every 1–2 months. For analysis of community dynamics, we used six days of netting for each net line collected during a period of <1 year from five time intervals: before isolation; 1984–1989 (2–6 years after isolation, hereafter ‘1985’ [the median year of these samples]); 1991–1992 (hereafter ‘1992’); 2000–2001 (hereafter ‘2000’); and 2007. One of the 100-ha fragments, 2303, was an exception to this pattern. It was isolated in 1990, with its first postisolation sample in 1992. We also have estimates of extinction and colonization in the absence of fragmentation effects. For four reserves that were later isolated, we analyzed two sets of preisolation samples separated by at least two years. We also sampled a continuous forest site in 1992 and 2000 following the protocol of 100-ha fragments (reserve 1501- see [34] for a map).

### Extinction and colonization parameter estimation

We used COMDYN4 [21], [22], [36] to estimate extinction and colonization parameters between sampling intervals (e.g. between 2000 and 2007). The approach used in COMDYN extends statistical methods used to estimate population size from capture-recapture approaches in single populations in which individuals differ in capture probability [37]. Further studies showed the same jackknife approach, but with species replacing individuals, to outperform other models to estimate species richness when detection probabilities differed among species (due to the species themselves, observer variation, or site effects [38]). The species richness estimator for a single community was then extended to describe extinction and colonization in communities over time [21], [22], [36].

Following the terminology of Nichols et al. [22], we refer to each day of netting as an occasion. Six occasions from a given fragment in a given year (e.g. 2000) comprise a sample, which is then compared with six occasions from the same fragment in the next sample (e.g. 2007). We assume violations of community closure within samples are negligible compared to variation across years [33]. COMDYN4 requires a list of species netted in each occasion, from which can also be calculated the number of species detected on exactly 1, 2, … 6 occasions within the sample, as well as the species common to both samples. From these data, COMDYN4 estimates the following parameters: phi, the proportion of species from a sample present in the next sample, equivalent to 1- (proportion of species extinct between two samples); and gamma, the proportion of species in the second sample that are present in the first sample, equivalent to 1- (proportion of species that turn over between samples). Hereafter we refer to 1-phi as extinction, and 1-gamma as colonization. COMDYN4 also calculates standard errors and 90% confidence intervals for parameters, as well as goodness of fit and heterogeneity of detection probabilities between samples. Although COMDYN4 estimates are relatively robust to goodness of fit violations (J.D. Nichols, personal communication), we reduced the number of occasions if goodness of fit >0.1 for a sample. For 1- and 10-ha fragments, we used five occasions for three samples, and four occasions for one sample. Both 100-ha fragments had less complete sampling, especially in 1992 and 2000; for these comparisons we used four or five occasions. No comparisons had heterogeneous capture probabilities (all p>0.1).

Throughout the analysis we emphasize colonization and extinction parameters rather than estimated species richness. Our repeated sampling allows us to focus on temporal turnover, rather than absolute numbers of species. Heterogeneity in sampling effort (number of nets) alone did not drive extinction and colonization parameter estimates, based on our analysis of preisolation samples that differed in number of nets but not in fragment size (see **Results- Extinction and colonization parameter estimates between time intervals**), so it is meaningful to compare these estimates across fragment size classes. We accept that ground-based sampling represents only a subset of the avifauna, those species that use the lowest stratum of the forest. On the other hand, turnover statistics reflect the pattern in that subset of species that sometimes gets caught in nets, and account for variation in capture probability.

The program CONTRAST can be used for hypothesis testing of parameter estimates from COMDYN4 [39]. This test provides a chi-squared statistic for heterogeneity among normally-distributed estimates based on the estimates and their standard errors. We used CONTRAST to test for fragment size effects on extinction and colonization estimates, to test for heterogeneity in extinction and colonization within fragments over time, and to test the effects of fragment reisolation on parameter estimates (see below). Before using CONTRAST, we tested for normality using the Wilk-Shapiro test; we did not proceed for data that were not normal (Wilk-Shapiro p>0.1).

Borders around most of the 1- and 10-ha fragments have been periodically cleared in a swath of 50–100 m. To consider this effect on extinction and colonization, we divided the samples from 1992, 2000, and 2007 into two groups depending on whether or not the border had been cut since the last sample. We then compared the extinction and colonization parameters for the two groups using CONTRAST. We expected fragment size also to affect these parameters [32], so we performed the analysis separately for 1- and 10-ha fragments. We could not include 100-ha fragments because they were never completely reisolated. A better design for this question would have been to make comparisons from the same fragment in intervals with and without border clearing. Unfortunately, reisolation was not sufficiently standardized among fragments to permit this model.

### Additional field sampling

COMDYN4 provides aggregate parameters for community dynamics, but no information on individual species. We examined extinction responses of individual species between preisolation and 2007 by examining whether species netted before isolation were present in the same fragments in 2007. To minimize the chance of overlooking species that were actually present, we expanded the standard net sampling protocol in 2007. First, we added an additional four nets on each of four borders of every fragment. Second, on each day of netting 2–4 experienced observers compiled a list of all birds seen or heard within the fragment or in an approximately 50 m band around the fragment (we included this band mostly because we were often unable to locate vocalizing birds precisely, especially in 1-ha fragments, when we heard them while we were otherwise occupied, such as handling birds). We used all of these sources (standard mist net sample, additional nets, and daily lists) to compile a list of birds detected in each fragment in 2007. For each species detected before isolation in each fragment, we used this list to assess if it was present or absent in 2007. Note that we make no assumption that species detected only in 2007 were actually missing before isolation; the additional sampling in 2007 was simply to minimize the chance of overlooking species that were present but not netted in the standard net sampling. Based on subsequent detailed surveys of the fragments, we are confident that very few species present in 2007 went undetected.

The BDFFP forest bird community has little influence of migrants. In forest understory, the only regular migrants are an intratropical migrant quail-dove and, less commonly, North American *Catharus* thrushes [40], [41], [42]. For the analysis here, we exclude migrants, as well as raptors and large ground birds (e.g. tinamous). Taxonomy follows Remsen et al. [43].

## Results

### Extinctions preisolation to 2007

**Figure 1:**
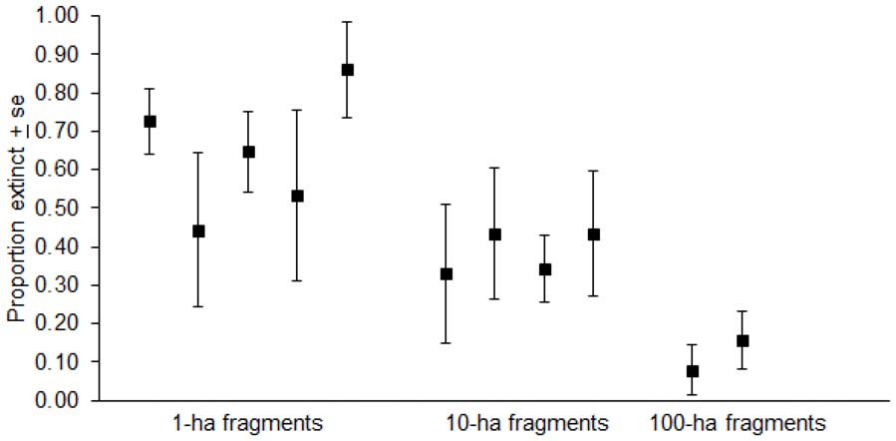
Proportion of preisolation species absent from each fragment in 2007. Extinction parameter estimates and standard errors from COMDYN4.

Between the preisolation and 2007 mist net samples we found strong area-dependent extinction rates, with mean extinction estimates from COMDYN4 of 44–84% in 1-ha fragments, 31–45% in 10-ha fragments, and 8–16% in 100-ha fragments over this approximately 25 year period (Figure 1).

We netted 101 species before isolation (Table S1). Based on the combined netting and additional surveys, 51 of these 101 species were absent from at least one fragment in 2007 where they had occurred before isolation. The proportion of species that went extinct at least once was strongly fragment- size dependent, with 59% of species going extinct from at least one 1-ha fragment, compared to 43% in 10-ha fragments and 12% in 100-ha fragments (Table S2). We considered the number of possible extinctions between isolation and 2007 based on the matrix of species x fragments in the preisolation sample (for example, a species present in two fragments before isolation would have two opportunities to go extinct). This approach showed significant heterogeneity in extinctions among size classes, with extinctions about three times higher in 10-ha fragments than in 100-ha fragments, and about five times higher in 1-ha fragments than in 100-ha fragments (G = 55.18, df = 2, p<0.001; Table S2).

### Extinction and colonization parameter estimates between time intervals

**Figure 2:**
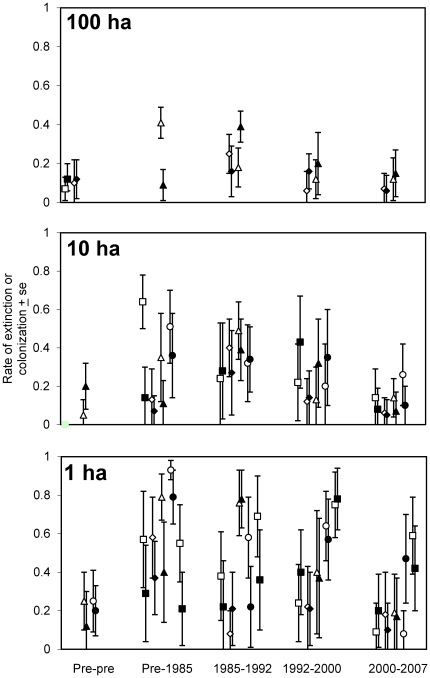
Extinction and colonization parameter estimates (± se). Each set of pairs of points of the same shape refers to an individual fragment, with the open symbol representing extinction and the filled symbol representing colonization for that interval. The 100-ha sample includes a preisolation-preisolation sample from a 100-ha plot in continuous forest sampled in 1992 and 2000 (squares). The 100-ha figure does not include a sample from preisolation – 1985 for plot 2303 (diamonds), which was not isolated until 1990.

COMDYN4 colonization and extinction estimates across all time intervals reveal two general trends across most fragments regardless of size class (Figure 2). First, by 2007, some 25 years after isolation, most fragments had extinction and colonization rates that were lower than they had been in the earlier years after isolation, suggesting that communities were increasingly stable over time. For fragments with two presisolation samples, the 2000–2007 samples generally showed comparable turnover in 20–25 year-old fragments as in two preisolation samples. The highest rate of extinction and the least overlap with colonization usually occurred in the earlier samples (either 1985 or 1991), followed by increased colonization and reduced extinction in 2000 and 2007. Second, by 2007 standard errors of colonization and extinction estimates broadly overlapped for most fragments, suggesting that these fragments had turnover of species since 2000, but little net change in number of species. Increasing overlap in extinction and colonization parameter estimates is especially apparent in 100-ha fragments, where for both fragments the estimates became increasingly similar until they were almost identical by 2007. It is important to reiterate that these COMDYN4 estimates are based on standardized and consistent sampling protocol beginning before isolation and continuing through 2007; additional survey techniques provided additional data for individual species, as described below in **Results- Extinction and colonization by individual species**, but were unrelated to the parameter estimates in Figure 2.

Fragment size clearly affected patterns of colonization and extinction (Figure 2). This was not due simply to unequal sampling effort (number of nets) across fragment size class; for sites with two preisolation samples the landscape setting remained the same (continuous forest), but the sampling effort differed (based on the size of the fragment to be isolated), but neither extinction nor colonization parameter estimates varied with number of nets (*X* ^2^<2.3, df = 2, p>0.30). This insignificant test result notwithstanding (e.g., [44]), parameter estimates were lower and had smaller standard errors with larger sampling effort, so we caution that comparisons across fragment size classes may most strongly reflect real biological processes, but perhaps also include more subtle effects of number of nets on COMDYN4. We attempted to standardize to 8 net samples to compare fragment size classes, but were unable to fit models without poor goodness of fit, especially in the first two intervals after isolation.

Highest extinction estimates come from 1-ha fragments, where extinction estimates exceeded 50% for every fragment in the first interval after isolation, and 1–3 fragments still had extinction rates >50% in the last three intervals (Figure 2). Fragment size effects on extinction could be tested for preisolation and for the first and second intervals. For the first two intervals after isolation, we found size-dependent differences in parameter estimates (interval 1: *X* ^2^ = 14.05, df = 2, p<0.001; interval 2: *X* ^2^ = 12.14, df = 2, p = 0.002). We could not test intervals 3 and 4 because parameter estimates were not normal (both Shapiro-Wilk p<0.1).

Colonization rates were also highest in 1-ha fragments (Figure 2). In 1-ha fragments, colonization approached 80% in at least one fragment in every interval except 2000–2007. In 10-ha fragments, no colonization parameter estimates exceeded 50%. Estimates were lower still for 100-ha fragments, with only two estimates >0.25; in fragment 3304, the first 100-ha fragment to be isolated, a 40% extinction rate in the first interval after isolation was followed with a 40% colonization rate in the following interval. Colonization estimates were only normal for preisolation and 1992–2000 (all other Shapiro-Wilk p<0.1). Colonization differed among fragment size classes in 1992–2000 (*X* ^2^ = 6.33, df = 2; p = 0.042).

Fragment size also appeared to affect variation in extinction and colonization estimates among fragments within time intervals (Figure 2). In 1-ha fragments, estimate standard errors often did not overlap among fragments, even in the 2000–2007 interval. In contrast, 10- and 100-ha fragments were more similar to each other, especially in the last two intervals.

### Which species went extinct?

To determine the species that accounted for the most extinctions between preisolation and 2007, we ranked species by their contribution to the total number of extinctions (Table 1). One species, *Myrmornis torquata,* was present in all 11 fragments before isolation but was absent everywhere in 2007. *Sclerurus caudacutus* and *Hylophylax naevius* were each detected in at least six fragments before isolation, but were absent in all fragments in 2007. These three species and *Neopipo cinnamomea*, which was detected in just one fragment before isolation, were the only species from the preisolation sample that were not detected anywhere in 2007. The remaining species in Table 1 were present in at least one fragment in 2007, typically one or both 100-ha fragments (Table S1). These species with the most extinctions were generally ubiquitous before isolation but absent from 1- and 10-ha fragments in 2007.

**Table 1:** The 15 species that accounted for the most extinctions from preisolation through 2007.

Species	Preisolation fragments	Proportion extinct	Proportion of total extinctions	Cumulative proportion
*Myrmornis torquata*	11	1.00	0.064	0.06
*Myrmotherula guttata*	11	0.82	0.052	0.12
*Cyphorhinus arada*	8	0.88	0.040	0.16
*Sclerurus caudacutus*	7	1.00	0.040	0.20
*Certhiasomus stictolaemus*	11	0.55	0.035	0.23
*Microbates collaris*	11	0.55	0.035	0.27
*Sclerurus rufigularis*	9	0.67	0.035	0.30
*Conopophaga aurita*	8	0.75	0.035	0.34
*Hylophylax naevius*	6	1.00	0.035	0.37
*Formicarius colma*	10	0.50	0.029	0.40
*Platyrinchus saturatus*	11	0.45	0.029	0.43
*Platyrinchus coronatus*	10	0.50	0.029	0.46
*Deconychura longicauda*	9	0.56	0.029	0.49
*Sclerurus mexicanus*	7	0.71	0.029	0.51
*Hylopezus macularius*	6	0.83	0.029	0.54

‘Proportion extinct’ is based on 2007 status from only fragments where the species was captured before isolation. ‘Proportion of total extinctions’ and ‘Cumulative proportion’ are based on the species' contribution to the total number of species x fragment extinctions from preisolation through 2007.

### Extinction and colonization by individual species

Species lists from 2000 and 2007 that include both netted birds and birds only seen or heard reveal 40 species from the 1992 mist net sample that went extinct or recolonized between 2000 and 2007, contributing to the patterns identified by COMDYN4 (Table 2). Of the species that were known to have gone extinct in at least one fragment between 1992 and 2000, 19 of 40 species recolonized at least one fragment between 2000 and 2007, and 23 species remained extinct in at least one fragment (two species did both). There were also 23 species that were known to be present in 2000 but were not detected by any method in the same fragments in 2007 (Table 3). Because we surveyed extensively by multiple methods in 2007, we think these species had gone extinct, rather than simply being overlooked.

**Table 2:** Status in 2007 of species that had gone extinct between 1992 and 2000.

	1-ha		10-ha		100-ha		Total	
Species	Extinct	Recolonize	Extinct	Recolonize	Extinct	Recolonize	Extinct	Recolonize
*Piaya melanogaster*			1	0			1	0
*Notharchus tectus*			0	1			0	1
*Malacoptila fusca*			1	0			1	0
*Galbula albirostris*			0	1			0	1
*Sclerurus rufigularis*			1	0			1	0
*Synallaxis rutilans*			1	0	1	0	2	0
*Automolus rubiginosus*	1	0					1	0
*Xenops minutus*	0	1					0	1
*Dendrocincla fuliginosa*	0	1					0	1
*Deconychura longicauda*	1	0					1	0
*Sittasomus griseicapillus*	1	0					1	0
*Dendrocolaptes picumnus*	0	1					0	1
*Lepidocolaptes albolineatus*			1	0			1	0
*Frederickena viridis*			1	0			1	0
*Myrmotherula guttata*	1	0			1	0	2	0
*Myrmotherula axillaris*	0	1					0	1
*Myrmotherula menetriesii*	1	0					1	0
*Schistocichla leucostigma*	1	0					1	0
*Pithys albifrons*	0	1					0	1
*Hylophylax naevius*	1	0					1	0
*Willisornis poecilinotus*			1	0			1	0
*Formicarius analis*					0	2	0	2
*Hylopezus macularius*					1	0	1	0
*Rhynchocyclus olivaceus*			1	0			1	0
*Platyrinchus saturatus*	1	0	0	1			1	1
*Terenotriccus erythrurus*	0	1	0	1			0	2
*Tyranneutes virescens*	0	1					0	1
*Pipra erythrocephala*	0	1					0	1
*Laniocera hypopyrra*	1	0	0	1			1	1
*Pachyramphus marginatus*	1	0					1	0
*Vireo olivaceus*	0	1					0	1
*Microcerculus bambla*			1	0			1	0
*Tachyphonus cristatus*					0	1	0	1
*Tachyphonus surinamus*	0	1					0	1
*Tangara punctata*			0	1			0	1
*Cyanerpes caeruleus*			1	0			1	0
*Saltator grossus*	0	2					0	2
*Arremon taciturnus*	0	1					0	1
*Cyanocompsa cyanoides*	3	0					3	0
*Phaeothlypis rivularis*					1	0	1	0
Total species	11	12	10	6	4	2	23	19

‘Extinct’ means the species continued to be absent from the fragment; ‘Recolonize’ means the species returned between 2000 and 2007. Species with no data may or may not have been present in 2000 or 2007, but their status in 1992 was not known with certainty (they were absent from the 1992 mist net sample, but not surveyed by other techniques in 1992).

**Table 3:** Species known to be present in 2000 that were not detected in the same fragment in 2007.

	Fragment size		
Species	1-ha	10-ha	100-ha
*Veniliornis cassini*	1		
*Sclerurus caudacutus*		1	
*Sclerurus mexicanus*		2	
*Certhiasomus stictolaemus*		2	
*Dendrocincla merula*		1	
*Hylexetastes perrotii*		1	
*Frederickena viridis*		2	1
*Thamnomanes ardesiacus*	1		
*Myrmotherula guttata*		1	
*Schistocichla leucostigma*			1
*Gymnopithys rufigula*		1	
*Willisornis poecilinotus*	1		
*Myrmornis torquata*			1
*Formicarius colma*		2	
*Conopophaga aurita*	1	1	
*Myrmothera companisona*		1	
*Platyrinchus saturatus*		2	
*Platyrinchus coronatus*		1	
*Myiobius barbatus*	1		
*Schiffornis turdina*		1	
*Microcerculus bambla*	2		
*Microbates collaris*	1	2	
*Cyanocompsa cyanoides*	1		
	9	21	3

Comparing known extinctions from preisolation through 2007 (Table 1) with known extinctions and recolonizations between 2000 and 2007 (Tables 2 and 3) reveals extinction dynamics for some species. For instance, 10 of the 15 high-extinction species from Table 1 went extinct from at least one fragment in the 2000–2007 interval (Table 3). Based on their presence in 2000, these species had persisted or recolonized between isolation and 2000. Six species from Table 1 had been extinct since the 1992–2000 interval in at least one fragment (Table 2). Unfortunately, although we know intervals when these species went extinct, we don't know if they were continually present following isolation, or if we documented extinction of recolonists. We do know, however, that about half the species that went extinct in the 1992–2000 interval had recolonized by 2007, and that recolonization occurred in all fragment size classes (Table 2). These results support the estimated extinction and colonization parameters based only on capture data (Figure 2).

### Effects of fragment reisolation

Both colonization and extinction parameter estimates varied with border clearing (Table 4). In 1-ha fragments, mean parameter estimates for both extinction and colonization were about twice as high in intervals with reisolation as in intervals without reisolation, although the estimates had wide ranges. Neither extinction nor colonization estimates for the entire sample were normal, but we were able to test estimates from just intervals 2 and 3. Extinction estimates were higher in reisolated fragments (*X* ^2^ = 7.6, df = 1, p = 0.006). For colonization, the difference approached significance (*X* ^2^ = 3.6, df = 1, p = 0.060). In 10-ha fragments, extinction and colonization estimates were normal for the entire sample, but there was no difference in extinction or colonization associated with fragment reisolation (both *X* ^2^<1.3, df = 1, p>0.27).

**Table 4:** Extinction and colonization parameter estimates partitioned by fragment size and reisolation status since the preceding sample, beginning with the 1985–1992 interval.

			Extinction		Colonization	
Size	Reisolated?	n	Mean	Range	Mean	Range
1	No	7	0.20	0.08–0.59	0.26	0.10–0.42
1	Yes	8	0.55	0.24–0.76	0.46	0.22–0.78
10	No	6	0.24	0.12–0.41	0.19	0.08–0.32
10	Yes	6	0.27	0.14–0.49	0.31	0.07–0.43

## Discussion

### Extinctions after 25 years

These results demonstrate a 25-year history of area-dependent extinctions in fragments. Turnover estimates from COMDYN4 and extensive field surveys in 2000 and 2007 provide complementary views of community change following isolation. The COMDYN4 analysis, which considers species present but not detected, estimated extinction of some 45–85% of species in 1-ha fragments, 30–45% in 10-ha fragments, and 10–15% in 100-ha fragments (Figure 1). Based on our surveys, we know which species accounted for many of these extinctions (Tables 1, S1).

When compared with previous whole-community analyses of extinctions in these fragments, our results suggest that the overall proportion of preisolation species that go extinct (or that are absent from a sample) changes little after about 10 years following isolation. Put another way, species richness did not continue to decline after about 10 years post-isolation. Ferraz et al. [33] used data through 1992 to estimate species loss; our results in 2007 do not show an increase in the proportion of preisolation species lost that would correspond to those estimates extended for another 15 years. In particular, models based only on species decay predicted many more extinctions than our results showed through 2007, especially for 10- and 100-ha fragments. On the other hand, the estimate in Ferraz et al. [33] that allowed colonization matched our results much better. Empirical capture and survey results showed a net increase in number of species between 1992 and 2000 [35], also demonstrating the role of colonization.

### Colonization

Including colonization dynamics provides a more complete view of temporal patterns than considering only extinction. As expected, adding additional sampling intervals revealed more extinctions and colonizations than considering only one long interval [45]. Even in the first interval after isolation, species colonized fragments (Figure 2). This could represent a carryover from the crowding effects immediately following isolation [17], [19], although colonizations have also been reported for forest islands isolated by comparable distance in Lago Guri, Venezuela [46]. In general, extinction rates exceeded colonization rates in the first or second intervals after isolation, through about 10 years, but the rates were more evenly matched in later intervals. Thus the relative stability of species richness from 1992–2007 was not because species did not go extinct- they did- it was because species were constantly colonizing, as we showed unambiguously for species that were well-surveyed in 2000 and 2007 (Table 2). Flux in species composition because of both extinction and colonization in fragments with non-equilibrial species richness conforms to both theory and classic observations from island biogeography [47], [48], and supports a view that the communities are not strongly structured by deterministic processes such as competition [49].

By 2007, most fragments had colonization and extinction rates comparable to preisolation samples. These preisolation comparisons were especially important in showing the flux expected due to a combination of both abiotic factors (number and placement of nets, and parameter estimation procedure) and local vagaries of species presence. We know that territories of some species appear and disappear from year to year, even in apparently suitable habitat in continuous forest [50]. Other species, however, would be expected to be stable in undisturbed forest (e.g., [51]). A challenge for the future will be to understand the extent of spatial and temporal dynamics of individual species in undisturbed forest and how this variation corresponds to patterns in fragmented landscapes.

In general, the landscapes around the PDBFF fragments have been steadily improving from the perspective of forest birds. Although active pastures remain in some areas, and some second growth has been cleared, much of the area that was originally deforested in the 1970s and 1980s has been abandoned to succession. This second growth is used by many species of forest birds [52], [53], [54]; see also [55], [56]. We know these changes strongly affect bird use of fragments; some species returned to fragments after second growth connected the fragments back to continuous forest [31]. Conversely, reisolation of fragments by even a narrow deforested band strongly affected capture rates [32].

Based on our previous analysis of capture rates [32], we expected a strong effect of reisolation on extinction and colonization, but the result was somewhat surprising. For 1-ha fragments, reisolation increased extinction rates, but also increased colonization rates (Table 4). This effect presumably explains some of the extreme heterogeneity among fragments even within the same time intervals (Figure 2). In 10-ha fragments, however, we found no effect of reisolation. Apparently, reisolation affects the number of birds using fragments more than it affects species richness. In 1-ha fragments, reisolation probably reduces the effective size of the fragments, making them more dynamic for both extinction and colonization.

### Vulnerable species

Our field surveys in 2007 allowed us to detect many species that were not netted. From these surveys, we determined the status of individual species present before isolation. We identified a subset of species that disappeared from fragments nearly everywhere they occurred before isolation, including even 100-ha fragments (Table 1). In general, these were the same species identified as vulnerable in previous analyses from our data, particularly ground- or near-ground-foraging insectivores [31], [35], [57]. Other studies of understory birds have also confirmed the vulnerability of species with similar traits, as well as the resilience of hummingbirds and frugivores [14], [55], [58], [59], [60]. The vulnerable species appear to be the most area-sensitive or least likely to move through second growth to recolonize fragments. Area sensitivity may be a particularly likely mechanism for vulnerability of some of the species in Table 1, such as *Sclerurus* spp., *Myrmornis torquata,* and *Cyphorhinus arada,* all of which require well more than 10-ha for normal territories [50], and thus would be expected to disappear from 1- or 10-ha fragments. Area sensitivity does not completely explain why these species were vulnerable in 100-ha fragments, however, nor does it explain why species with 5–10 ha territories, such as *Conopophaga aurita*, *Formicarius colma*, and *Platyrinchus* spp. [42], [50] disappeared from 10-ha fragments. We also identified a large suite of species, amounting to about half the species in 1- and 10-ha fragments, that sometimes went extinct, but also regularly recolonized between 2000–2007 (Table 2). These species may be vulnerable to fragmentation, but are also capable of recolonization, even of 1-ha fragments.

Although we identified a subset of species that are vulnerable even in 100-ha fragments, and almost never occurred after isolation in 1- or 10-ha fragments, most species occurred at least occasionally in 1- and 10-ha fragments. Communities in these smaller fragments were highly dynamic, however, with turnover at least equal to preisolation through the entire sampling period. This suggests that fragment communities were not reduced by extinction to some stable subset of preisolation species, but that a large pool of species regularly appeared and disappeared from fragments, probably due to colonization from nearby continuous forest. This turnover means that communities differ more among smaller fragments than among larger fragments, a result also observed from snapshot samples of birds in eastern Amazonian fragments and from the BDFFP fragments through 1992 [31], [61].

### Extinction debt

Studies with birds in fragments have typically shown fewer extinctions than expected based on area effects (reviewed in [8], [15]). Alternative explanations for this observation include a time lag between isolation and extinctions, implying that the extinctions will eventually occur (an extinction debt; [7]), or resilience of species in fragments even in the face of area reduction, with the far different implication that additional extinctions will not necessarily occur. An excess of species over the prediction of the species-area relationship could be through repeated recolonization [62], or due to a fundamental failure of the species-area relationship for fragments in landscapes that retain a significant proportion of original habitat (a fragmentation threshold; [3], [6], [63]). One approach we could use to consider these scenarios for our 25 years of post-isolation data would be to calculate the expected number of species to be lost for each size class following isolation [7]. Unfortunately, this approach would be problematic because it requires assumptions about the slope of the species-area curve, uses incomplete species lists before isolation, and is conceptually flawed [64]. Even without estimating expected extinctions, however, our results suggest that the net number of species in any of the fragments we studied is unlikely to decline further, implying that significant extinction debt does not remain for our fragments. We base this conclusion on our observation that colonization and extinction are generally in balance as of 20–25 years after isolation (Figure 2, Table 2).

Of course, species were lost in an area-dependent pattern, and that pattern has taken up to 25 years to play out (Figure 1; see also [35]). As of 2007, about half of all forest species captured before isolation still occur in some 1-ha fragments, and less than 10% have been lost from 100-ha fragments. Apparently, only a small subset of species is truly vulnerable throughout this landscape, as identified in Table 2. In landscapes like ours, with the potential for recolonization, it may be generally inaccurate to forecast extinctions assuming indefinite continuation of the rate exhibited soon after isolation by the most vulnerable species, although this might be expected in more heavily deforested landscapes [65]. Certainly the pattern of species richness in fragments needs to be considered in a landscape context, as recently illustrated for small mammals in Brazilian Atlantic forest fragments [6].

Despite their high colonization, we believe that many species present in small fragments like ours have little hope of demographic stability. This is not due only to area effects reducing potential population size in fragments (e.g. [46]), but also to species rarity- a manifestion of the Allee effect (e.g., [66]). That is, rare species may be unlikely to have two individuals that could potentially form a pair colonize a fragment in the same time window, an effect that could be common across taxa and landscapes (e.g., [67]). We suggest that high turnover in our small fragments often represents single colonists that arrive, are unable to find a mate, and leave or die without reproducing. This would be consistent with our turnover results, with the low abundance of some species and guilds in fragments, especially in the first years after isolation when the matrix was least hospitable [31], [32], and with new data showing disproportionate numbers of immature birds in fragments (E.I. Johnson et al., unpublished). Based on colonization even in the first years after isolation, only a few years of second growth development is necessary for forest birds to occasionally pass through the matrix (see also [68]). As the matrix matures, our challenge is to identify when improving habitat quality in second-growth landscapes allows small fragments to move beyond being population sinks occupied only by surplus individuals produced in nearby continuous forest [69], [70].

Our results suggest that extreme extinction scenarios (e.g., [71], [33]) are not occurring in our landscapes. This is good news for conservation; it implies that most Amazonian primary forest bird species can use a network of second growth and small fragments. At the same time, our landscape setting includes vast continuous forest; it remains unclear if secondary forest and fragments alone could support viable populations of Amazonian forest birds (*sensu* [27], [28]).

## Supporting Information

Table S1 All species captured before isolation and their status in 2007. For each fragment size class, ‘Pre’ is the number of fragments where the species was detected preisolation, and ‘2007’ is the number of those same fragments where it was detected by any means in 2007 (thus a fragment where a species was detected only in 2007 does not get counted). ‘2007 total’ includes all fragments where the species was detected in 2007, regardless of whether it was captured before isolation. Taxonomy and sequence follow Remsen et al. 2010.
(DOC)

Table S2 Extinctions between preisolation and 2007 by fragment size class. ‘Extinct species’ and ‘Proportion of species extinct’ include all species that went extinct in any fragment of that size class, even if the species persisted in other fragments of the same size class. ‘Proportion of possible extinctions’ represents the total number of species x fragment combinations from before isolation that were not present in 2007.
(DOC)
