# Supplementary material for: Understory Bird Communities in Amazonian Rainforest Fragments: Species Turnover through 25 Years Post-Isolation in Recovering Landscapes
Source: PLoS One. 2011 Jun 22;6(6):e20543. doi: 10.1371/journal.pone.0020543 (PMC3120763; doi:10.1371/journal.pone.0020543)
Supplement: Table S1 — All species captured before isolation and their status in 2007. For each fragment size class, ‘Pre’ is the number of fragments where the species was detected preisolation, and ‘2007’ is the number of those same fragments where it was detected by any means in 2007 (thus a fragment where a species was detected only in 2007 does not get counted). ‘2007 total’ includes all fragments where the species was detected in 2007, regardless of whether it was captured before isolation. Taxonomy and sequence follow Remsen et al. 2010. (DOC) [file pone.0020543.s001.doc]

**Table S1. All species captured before isolation and their status in 2007.** For each fragment size class, ‘Pre’ is the number of fragments where the species was detected preisolation, and ‘2007’ is the number of those same fragments where it was detected by any means in 2007 (thus a fragment where a species was detected only in 2007 does not get counted). ‘2007 total’ includes all fragments where the species was detected in 2007, regardless of whether it was captured before isolation. Taxonomy and sequence follow [43].

|  |  |  |  |  |  |  |  |  |  |  |
| --- | --- | --- | --- | --- | --- | --- | --- | --- | --- | --- |
|  | 1-ha | |  | 10-ha | |  | 100-ha | |  | 2007 |
| Species | Pre | 2007 |  | Pre | 2007 |  | Pre | 2007 |  | total |
| *Phaethornis superciliosus* | 4 | 4 |  | 4 | 4 |  | 2 | 2 |  | 11 |
| *Phaethornis bourcieri* | 4 | 4 |  | 4 | 4 |  | 2 | 2 |  | 11 |
| *Campylopterus largipennis* |  |  |  | 1 | 1 |  | 2 | 2 |  | 9 |
| *Florisuga mellivora* |  |  |  |  |  |  | 1 | 1 |  | 9 |
| *Thalurania furcata* | 4 | 4 |  | 4 | 4 |  | 2 | 2 |  | 11 |
| *Hylocharis sapphirina* |  |  |  |  |  |  | 1 | 1 |  | 2 |
| *Heliothryx auritus* |  |  |  |  |  |  | 1 | 1 |  | 9 |
| *Trogon rufus* | 1 | 1 |  |  |  |  | 1 | 1 |  | 7 |
| *Momotus momota* | 4 | 4 |  | 4 | 4 |  | 2 | 2 |  | 11 |
| *Chloroceryle inda* |  |  |  | 1 | 0 |  | 1 | 1 |  | 1 |
| *Chloroceryle aenea* |  |  |  |  |  |  | 1 | 1 |  | 1 |
| *Malacoptila fusca* | 2 | 0 |  | 3 | 2 |  | 2 | 2 |  | 6 |
| *Monasa atra* |  |  |  |  |  |  | 1 | 1 |  | 11 |
| *Galbula albirostris* | 3 | 2 |  | 2 | 2 |  | 2 | 2 |  | 10 |
| *Jacamerops aureus* |  |  |  | 1 | 1 |  |  |  |  | 10 |
| *Campephilus rubricollis* |  |  |  |  |  |  | 1 | 1 |  | 9 |
| *Sclerurus mexicanus* | 3 | 0 |  | 2 | 0 |  | 2 | 2 |  | 2 |
| *Sclerurus rufigularis* | 4 | 0 |  | 3 | 1 |  | 2 | 2 |  | 3 |
| *Sclerurus caudacutus* | 2 | 0 |  | 3 | 0 |  | 2 | 0 |  | 0 |
| *Synallaxis rutilans* | 2 | 0 |  | 1 | 0 |  | 1 | 1 |  | 1 |
| *Philydor erythrocercum* | 3 | 1 |  | 2 | 1 |  |  |  |  | 5 |
| *Philydor pyrrhodes* |  |  |  | 1 | 1 |  | 2 | 2 |  | 4 |
| *Automolus infuscatus* | 4 | 1 |  | 4 | 4 |  | 2 | 2 |  | 8 |
| *Automolus rubiginosus* | 2 | 0 |  | 3 | 1 |  | 2 | 2 |  | 3 |
| *Automolus ochrolaemus* | 3 | 3 |  | 3 | 3 |  | 2 | 2 |  | 11 |
| *Xenops minutus* | 5 | 5 |  | 4 | 4 |  | 2 | 2 |  | 11 |
| *Certhiasomus stictolaemus* | 5 | 2 |  | 4 | 1 |  | 2 | 2 |  | 5 |
| *Dendrocincla fuliginosa* | 5 | 4 |  | 3 | 3 |  | 2 | 2 |  | 10 |
| *Dendrocincla merula* | 5 | 2 |  | 3 | 2 |  | 2 | 2 |  | 7 |
| *Deconychura longicauda* | 4 | 0 |  | 3 | 2 |  | 2 | 2 |  | 6 |
| *Sittasomus griseicapillus* | 1 | 1 |  |  |  |  | 2 | 2 |  | 10 |
| *Glyphorynchus spirurus* | 5 | 5 |  | 4 | 4 |  | 2 | 2 |  | 11 |
| *Hylexetastes perrotii* | 2 | 2 |  | 2 | 2 |  | 1 | 1 |  | 5 |
| *Dendrocolaptes certhia* | 3 | 3 |  | 3 | 1 |  | 2 | 2 |  | 11 |
| *Dendrocolaptes picumnus* | 1 | 0 |  | 3 | 2 |  | 1 | 1 |  | 7 |
| *Xiphorhynchus pardalotus* | 5 | 5 |  | 4 | 4 |  | 2 | 2 |  | 11 |
| *Campylorhamphus procurvoides* | 4 | 0 |  | 3 | 3 |  | 2 | 2 |  | 5 |
| *Cymbilaimus lineatus* |  |  |  |  |  |  | 1 | 1 |  | 8 |
| *Frederickena viridis* | 3 | 0 |  | 3 | 0 |  | 1 | 1 |  | 1 |
| *Thamnophilus murinus* | 1 | 1 |  | 4 | 4 |  | 2 | 2 |  | 11 |
| *Thamnomanes ardesiacus* | 5 | 2 |  | 4 | 4 |  | 2 | 2 |  | 8 |
| *Thamnomanes caesius* | 4 | 4 |  | 3 | 3 |  | 2 | 2 |  | 10 |
| *Epinecrophylla gutturalis* | 5 | 3 |  | 4 | 4 |  | 2 | 2 |  | 9 |
| *Myrmotherula guttata* | 5 | 1 |  | 4 | 0 |  | 2 | 1 |  | 2 |
| *Myrmotherula axillaris* | 3 | 3 |  | 3 | 3 |  | 2 | 2 |  | 11 |
| *Myrmotherula longipennis* | 5 | 4 |  | 4 | 3 |  | 2 | 2 |  | 9 |
| *Myrmotherula menetriesii* | 4 | 1 |  | 3 | 3 |  | 2 | 2 |  | 8 |
| *Hypocnemis cantator* | 2 | 2 |  | 4 | 4 |  | 2 | 2 |  | 11 |
| *Percnostola rufifrons* | 4 | 4 |  | 3 | 3 |  | 2 | 2 |  | 11 |
| *Schistocichla leucostigma* |  |  |  | 1 | 1 |  | 1 | 1 |  | 2 |
| *Myrmeciza ferruginea* | 4 | 3 |  | 2 | 2 |  | 2 | 2 |  | 7 |
| *Myrmeciza atrothorax* |  |  |  | 1 | 0 |  | 1 | 1 |  | 1 |
| *Pithys albifrons* | 5 | 5 |  | 4 | 4 |  | 2 | 2 |  | 11 |
| *Gymnopithys rufigula* | 4 | 3 |  | 4 | 3 |  | 2 | 2 |  | 9 |
| *Hylophylax naevius* | 1 | 0 |  | 4 | 0 |  | 1 | 0 |  | 0 |
| *Willisornis poecilinotus* | 5 | 2 |  | 4 | 3 |  | 2 | 2 |  | 7 |
| *Formicarius colma* | 5 | 2 |  | 3 | 1 |  | 2 | 2 |  | 6 |
| *Formicarius analis* | 3 | 0 |  |  |  |  | 1 | 1 |  | 5 |
| *Myrmornis torquata* | 5 | 0 |  | 4 | 0 |  | 2 | 0 |  | 0 |
| *Grallaria varia* |  |  |  | 1 | 1 |  | 1 | 1 |  | 7 |
| *Hylopezus macularius* | 3 | 0 |  | 1 | 0 |  | 2 | 2 |  | 2 |
| *Conopophaga aurita* | 4 | 0 |  | 2 | 0 |  | 2 | 2 |  | 2 |
| *Corythopis torquatus* | 2 | 0 |  | 3 | 1 |  | 2 | 2 |  | 3 |
| *Mionectes macconnelli* | 5 | 5 |  | 4 | 4 |  | 2 | 2 |  | 11 |
| *Hemitriccus zosterops* | 1 | 1 |  | 1 | 1 |  | 2 | 2 |  | 7 |
| *Rhynchocyclus olivaceus* | 3 | 2 |  | 3 | 2 |  | 2 | 2 |  | 8 |
| *Tolmomyias assimilis* | 1 | 1 |  | 1 | 1 |  |  |  |  | 10 |
| *Platyrinchus saturatus* | 5 | 2 |  | 4 | 2 |  | 2 | 2 |  | 6 |
| *Platyrinchus coronatus* | 4 | 0 |  | 4 | 3 |  | 2 | 2 |  | 6 |
| *Platyrinchus platyrhynchos* | 1 | 0 |  | 2 | 1 |  | 1 | 1 |  | 3 |
| *Onychorhynchus coronatus* |  |  |  | 4 | 2 |  | 2 | 2 |  | 4 |
| *Myiobius barbatus* | 4 | 4 |  | 4 | 4 |  | 2 | 2 |  | 8 |
| *Terenotriccus erythrurus* | 2 | 2 |  | 3 | 3 |  | 2 | 2 |  | 11 |
| *Neopipo cinnamomea* |  |  |  |  |  |  | 1 | 0 |  | 0 |
| *Ramphotrigon ruficauda* |  |  |  | 1 | 1 |  |  |  |  | 2 |
| *Rhytipterna simplex* | 1 | 1 |  |  |  |  | 1 | 1 |  | 10 |
| *Attila spadiceus* |  |  |  |  |  |  | 1 | 1 |  | 11 |
| *Phoenicircus carnifex* | 1 | 1 |  | 1 | 1 |  | 1 | 1 |  | 9 |
| *Lipaugus vociferans* | 1 | 1 |  |  |  |  | 1 | 1 |  | 11 |
| *Corapipo gutturalis* |  |  |  | 2 | 2 |  | 2 | 2 |  | 11 |
| *Lepidothrix serena* | 5 | 5 |  | 2 | 2 |  | 2 | 2 |  | 11 |
| *Pipra pipra* | 5 | 5 |  | 4 | 4 |  | 2 | 2 |  | 11 |
| *Pipra erythrocephala* | 1 | 1 |  | 4 | 4 |  | 2 | 2 |  | 11 |
| *Schiffornis turdina* | 5 | 1 |  | 4 | 2 |  | 2 | 2 |  | 5 |
| *Laniocera hypopyrra* |  |  |  |  |  |  | 1 | 1 |  | 3 |
| *Piprites chloris* |  |  |  |  |  |  | 1 | 1 |  | 10 |
| *Pheugopedius coraya* |  |  |  |  |  |  | 1 | 1 |  | 11 |
| *Microcerculus bambla* | 4 | 1 |  | 3 | 1 |  | 2 | 2 |  | 5 |
| *Cyphorhinus arada* | 3 | 0 |  | 3 | 0 |  | 2 | 1 |  | 1 |
| *Turdus albicollis* | 5 | 4 |  | 4 | 4 |  | 2 | 2 |  | 10 |
| *Microbates collaris* | 5 | 1 |  | 4 | 2 |  | 2 | 2 |  | 5 |
| *Hylophilus muscicapinus* |  |  |  |  |  |  | 1 | 1 |  | 9 |
| *Hylophilus ochraceiceps* | 4 | 3 |  | 4 | 4 |  | 2 | 2 |  | 10 |
| *Tachyphonus cristatus* |  |  |  | 1 | 1 |  | 1 | 1 |  | 9 |
| *Tachyphonus surinamus* | 3 | 3 |  | 3 | 3 |  | 2 | 2 |  | 11 |
| *Tangara chilensis* |  |  |  |  |  |  | 1 | 1 |  | 1 |
| *Coereba flaveola* |  |  |  | 1 | 1 |  | 1 | 1 |  | 9 |
| *Saltator grossus* |  |  |  | 1 | 1 |  |  |  |  | 9 |
| *Arremon taciturnus* |  |  |  | 1 | 0 |  |  |  |  | 4 |
| *Cyanocompsa cyanoides* |  |  |  | 2 | 2 |  | 2 | 2 |  | 6 |
| *Phaeothlypis rivularis* |  |  |  |  |  |  | 1 | 1 |  | 2 |
|  |  |  |  |  |  |  |  |  |  |  |
