# Supplementary material for: Understory Bird Communities in Amazonian Rainforest Fragments: Species Turnover through 25 Years Post-Isolation in Recovering Landscapes
Source: PLoS One. 2011 Jun 22;6(6):e20543. doi: 10.1371/journal.pone.0020543 (PMC3120763; doi:10.1371/journal.pone.0020543)
Supplement: Table S2 — Extinctions between preisolation and 2007 by fragment size class. ‘Extinct species’ and ‘Proportion of species extinct’ include all species that went extinct in any fragment of that size class, even if the species persisted in other fragments of the same size class. ‘Proportion of possible extinctions’ represents the total number of species x fragment combinations from before isolation that were not present in 2007. (DOC) [file pone.0020543.s002.doc]

**Table S2. Extinctions between preisolation and 2007 by fragment size class.** ‘Extinct species’ and ‘Proportion of species extinct’ include all species that went extinct in any fragment of that size class, even if the species persisted in other fragments of the same size class. ‘Proportion of possible extinctions’ represents the total number of species x fragment combinations from before isolation that were not present in 2007.

|  |  |  |  |
| --- | --- | --- | --- |
|  |  |  |  |
|  | Fragment size class | | |
|  | 1-ha | 10-ha | 100-ha |
| Preisolation species | 71 | 81 | 95 |
| Extinct species | 42 | 35 | 11 |
| Proportion of species extinct | 0.59 | 0.43 | 0.12 |
| Proportion of possible extinctions | 0.40 | 0.27 | 0.08 |
